# Why segment the maxilla between laterals and canines?

**DOI:** 10.1590/2177-6709.21.1.110-125.sar

**Published:** 2016

**Authors:** Lucas Senhorinho Esteves, Jean Nunes dos Santos, Steven M. Sullivan, Luana Maria Rosário Martins, Carolina Ávila

**Affiliations:** 1 Coordinator of the Center for Dentofacial Deformity of Hospital Santa Casa de Misericórdia, Salvador, Bahia, Brazil; 2 Associate Professor, Universidade Federal da Bahia (UFBA ), Department of Propaedeutics and Integrated Clinic, School of Dentistry, Salvador, Bahia, Brazil; 3 Full Professor and Chairman, University of Oklahoma, Department of Oral and Maxillofacial Surgery, College of Dentistry, Oklahoma City, OK, USA; 4 Undergraduate, Universidade Federal da Bahia (UFBA ), School of Dentistry, Salvador, Bahia, Brazil; 5 Staff of the Center for Dentofacial Deformity of Hospital Santa Casa de Misericórdia, Salvador, Bahia, Brazil

**Keywords:** Anterior segmental maxillary osteotomy, Orthognathic surgery, Dentoskeletal deformities

## Abstract

**Introduction:**

Maxillary surgery on a bone segment enables movement in the sagittal and vertical planes. When performed on multiple segments, it further provides movement in the transverse plane. Typical sites for interdental osteotomies are between laterals and canines, premolars and canines, or between incisors. Additionally, osteotomies can be bilateral, unilateral or asymmetric. The ability to control intercanine width, buccolingual angulation of incisors, and correct Bolton discrepancy are some of the advantages of maxillary segmentation between laterals and canines.

**Objective:**

This article describes important features to be considered in making a clinical decision to segment the maxilla between laterals and canines when treating a dentoskeletal deformity. It further discusses the history of this surgical approach, the indications for its clinical use, the technique used to implement it, as well as its advantages, disadvantages, complications and stability. It is therefore hoped that this paper will contribute to disseminate information on this topic, which will inform the decision-making process of those professionals who wish to make use of this procedure in their clinical practice.

**Conclusions:**

Segmental maxillary osteotomy between laterals and canines is a versatile technique with several indications. Furthermore, it offers a host of advantages compared with single-piece osteotomy, or between canines and premolars.

## Abstract (pt)

**Introdução:**

a cirurgia de maxila em um segmento ósseo possibilita sua movimentação nos planos sagital e vertical. Quando realizada em múltiplos segmentos, contempla também o plano transversal. A localização das osteotomias interdentárias pode ser: entre incisivos laterais e caninos, entre pré-molares e caninos, ou entre os incisivos centrais - podendo ser bilateral, unilateral ou assimétrica. A possibilidade de controle da distância intercaninos, da angulação vestibulolingual dos incisivos e da correção da discrepância de Bolton é uma das vantagens da segmentação da maxila entre incisivos laterais e caninos.

**Objetivo:**

o presente artigo descreve aspectos importantes, no tratamento das deformidades dentoesqueletais, a serem considerados na decisão clínica de segmentar-se a maxila entre os incisivos laterais e os caninos. Serão discutidas, também, a história dessa abordagem cirúrgica, as indicações para seu emprego clínico, a técnica para sua utilização, bem como suas vantagens, desvantagens, complicações e estabilidade. Desse modo, espera-se contribuir para o aumento das informações disponíveis sobre o assunto, subsidiando a decisão dos profissionais que queiram considerar o uso dessa técnica em suas atividades clínicas.

**Conclusão:**

a osteotomia segmentar de maxila entre os incisivos laterais e os caninos corresponde a uma técnica versátil com diversos tipos de indicações, e apresenta uma série de vantagens, quando comparada com as osteotomias em um segmento ou entre caninos e pré-molares.

## INTRODUCTION

Cohn-Stock was the first to describe anterior segmental maxillary osteotomy in 1921. Since then, several changes have been made to this surgical approach and new osteotomy models have emerged.^1^^,^ 2 Currently, maxillary surgery is a routine procedure for the correction of dentofacial deformities, and can be performed in one or multiple bone segments.^3^^,^ 4 Venugoplan et al^5^ found, after studying the number and types of procedures performed on patients hospitalized for orthognathic surgery in the United States, that maxillary segmentation is the most frequently performed procedure, involving 45,8% of the cases.^5^

**Figure 1:**
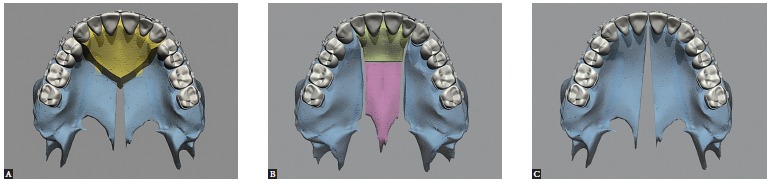
- Occlusal view illustrative of segmental maxillary osteotomy: A) Interdental osteotomies between canines and premolars; B) interdental osteotomies between laterals and canines; C) interdental osteotomy between central incisors.

Maxillary surgery on a bone segment enables movement in the sagittal and vertical planes. When performed in multiple segments, it also comprises the transverse plane. It is therefore touted as a rather versatile technique.^6^ Two or three bone segments can be used. Moreover, interdental osteotomy can be performed in the following sites: between laterals and canines, between premolars and canines (with or without premolar extractions), or between incisors. It can be bilateral, unilateral or asymmetric^7^ (Fig 1). 

Segmental maxillary surgery between canines and premolars, or between central incisors, is cited by many authors who report its advantages, disadvantages, complications and stability.^3^^,^ 8 ^,^ 9 ^,^ 10 Very few articles in the literature address the technique of segmental maxillary osteotomy between laterals and canines. Reyneke^9^ and Wolford et al^10^ cited the technique and emphasized some of its advantages, such as: management of intercanine width, and of the curves of Spee and Wilson; control of incisor buccolingual angulation; less orthodontic mechanics in the postoperative phase; and greater overall ease.

This surgical technique is indicated for the treatment of maxillary protrusion of which repositioning with orthodontic treatment alone is not feasible due to substantial tooth movement and potential damage to the periodontium.^3^^,^ 8 It can correct multiplanar maxillary deformities within a single surgical stage, such as in the following conditions: transverse maxillary expansion concurrently with vertical and sagittal positioning of incisors; anterior open bite correction specifically indicated to speed up orthodontic treatment time;^3^^,^ 11 ^,^ 12 and to correct tooth size discrepancy.^10^

Although considerable advances in the stability and predictability of maxillary surgery have been made over the years, complications can still occur, such as bone necrosis,^4^ oronasal and sinus fistula, tooth devitalization and periodontal defects.^1^^,^ 8 ^,^ 11

Therefore, based on the very scarce scientific literature available for this technique and the clinical experience of the authors, this article aims to address key issues to be considered when making a clinical decision to segment the maxilla between laterals and canines in the treatment of dentoskeletal deformities. It further discusses the history of this surgical approach, indications for its clinical use and the recommended technique as well as its advantages, disadvantages, complications and stability. It is therefore hoped that this paper will contribute to disseminate information on this topic, which will inform the decision-making process of those professionals who wish to make use of this procedure in their clinical practice.

## HISTORY

Although segmental maxillary osteotomy is currently employed in many treatment centers for dentofacial deformities, its development has been gradual and characterized by a long history of surgical techniques. Von Langenbeck described the use of horizontal osteotomies for the first time in 1859, and used this technique in 1861 to resect a patient's maxilla.^13^ His pioneering efforts were followed by colleagues around the world, which has led to the emergence of various changes and new techniques.

In 1867, Cheever described the Le Fort I maxillary technique involving mandibular displacement to facilitate access to the nasopharyngeal region with the purpose of resecting a tumor.^13^ In 1921, Cohn-Stock performed the first anterior segmental maxillary osteotomy to treat a skeletal maxillary protrusion. Despite improvements in occlusion, this procedure compromised facial esthetics due to an excessive retraction of anterior teeth.^14^ This approach was the starting point for the development of new techniques.^13^^-^ 16 In the 1980s, as a result of these developments, the increased flexibility of different types of osteotomy, advances in Orthodontics and orthognathic surgery, these techniques have become a standard procedure for correction of dentofacial deformities in the three dimensions.^17^^,^ 18

## INDICATIONS OF MAXILLARY SEGMENTATION BETWEEN LATERALS AND CANINES

Preoperative orthodontic goals play a major role in determining when premolar extraction will be required, when the curves of Spee, either marked or reverse, will be leveled orthodontically or surgically, when intra and inter-arches orthodontic procedures will be required to obtain appropriate dental positions, and how the maxillomandibular transverse relationship will be addressed.^19^ The indication to segment the maxilla between laterals and canines should be established during the phase of orthodontic and surgical planning (Table 1).

**Table 1:** - Summary of indications to segment the maxilla between laterals and canines.

Indications to segment the maxilla between laterals and canines.
1- Poor transverse relationship of the maxilla and control of intercanine width.
2- Correcting Bolton discrepancy.
3- Controlling incisor buccolingual angulation.
4- An easier technique.

### Poor transverse relationship of the maxilla and 

### control of intercanine width

**Figure 2:**
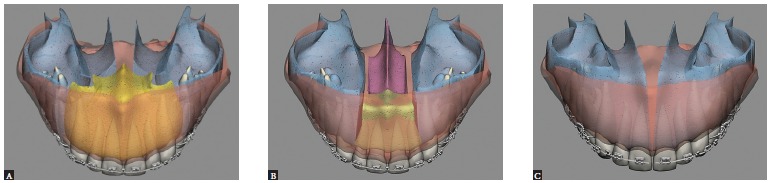
- Upper view illustrative of segmental maxillary osteotomy: A) osteotomies between canines and premolars; B) osteotomies between laterals and canines; C) osteotomy between central incisors.

Maxillary expansion surgery by means of interdental osteotomies yields a good transverse relationship between a hypoplastic maxilla and the mandible. However, segmental maxillary osteotomy technique applied between canines and premolars does not allow manipulation of intercanine width, given that the canines are located in the same bone block (Fig 2A). Correction of maxillomandibular transverse discrepancy in the region of canines would not be feasible. 

**Figure 3:**
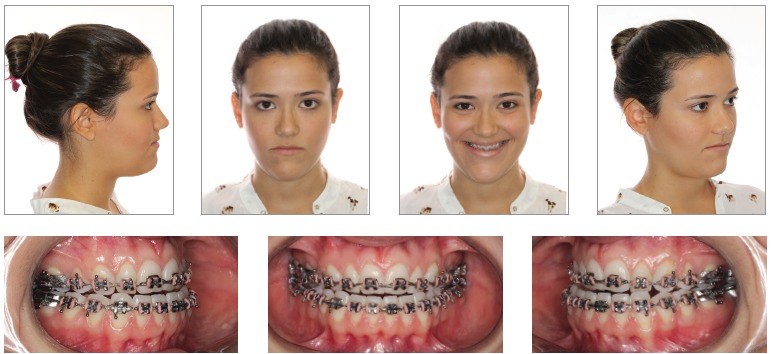
- Preoperative facial and intraoral photographs.

**Figure 4:**
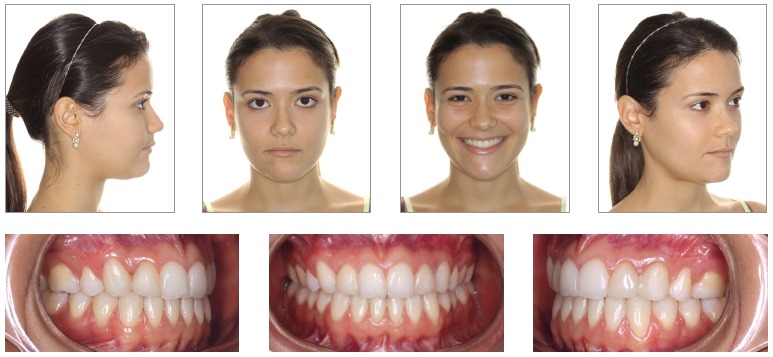
- Postoperative facial and intraoral photographs.

Once the technique is implemented between laterals and canines, it becomes possible to manipulate the intercanine width (Fig 2B). This approach favors changes in the torques of bone segments, in the curve of Wilson, and correction of transverse maxillomandibular discrepancy in the regions of molars, premolars and canines. When osteotomy is performed between central incisors, the intercanine width could be manipulated, but surgical correction of torque control of the posterior segments would be harder to implement, since each segment would comprise incisors, canines, premolars and molars and these teeth have different torques^9^ (Figs 2C, 3, 4). 

### Correcting Bolton discrepancy

Size discrepancy in individual teeth or groups of teeth may be associated with the emergence of changes in occlusion. For maxillary teeth to occlude properly and harmoniously with their mandibular antagonists, there must be adequate proportionality between different tooth sizes.^20^ Pizzol et al^21^ reported an average 90% of presence of Bolton discrepancy in patients with dentoskeletal deformities. When caused by excessive anteroinferior dental volume, this discrepancy can be corrected in several ways: Selective interproximal dental stripping, changes in the buccolingual or mesiodistal angulation of anterior teeth, mandibular incisor extraction, or by creating space in the upper jaw between laterals and canines. These spaces can be created through orthodontic mechanics, such as the use of springs and changes in the buccolingual angulation of incisors, or by maxillary surgical segmentation between laterals and canines.^10^

**Figure 5:**
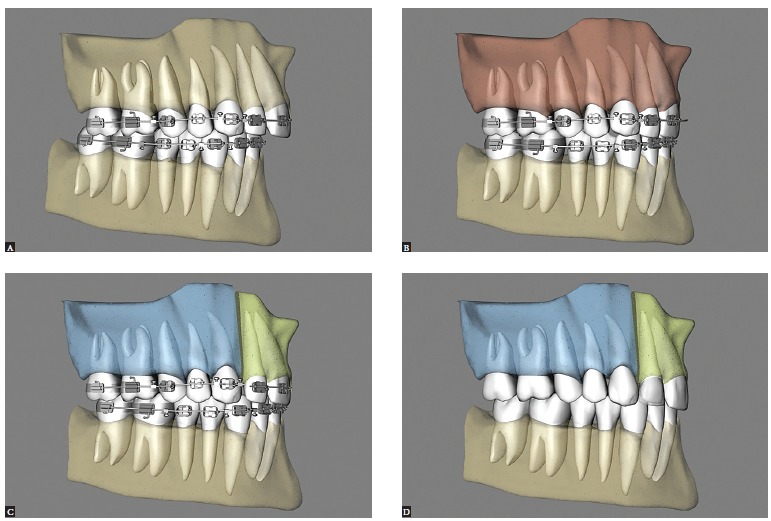
- Lateral view illustrative of clinical case with Bolton discrepancy and excess lower dental volume: A) Preoperative clinical condition showing a Class II sagittal relationship and mesiodistal size deficiency of maxillary teeth (smaller laterals). B) Single-piece maxillary surgery: canine Class II sagittal relationship due to Bolton discrepancy. C) Maxillary surgery in three segments: canine Class I sagittal relationship with presence of diastema between laterals and canines to correct Bolton discrepancy. D) Three-segment maxillary surgery in esthetic stage where the diastema has been closed through indirect restoration.

**Figure 6:**
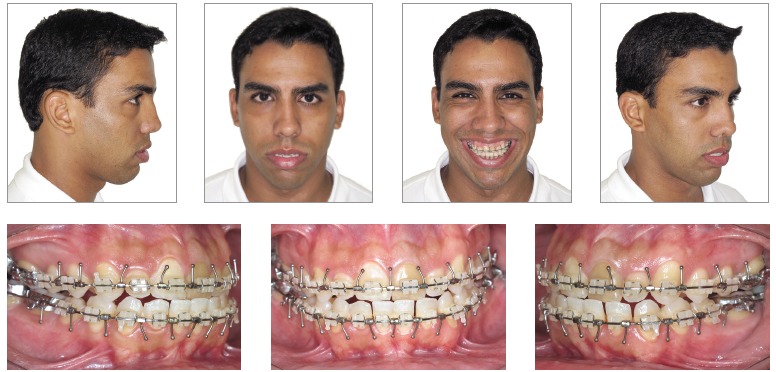
- Preoperative facial and intraoral photographs.

**Figure 7:**
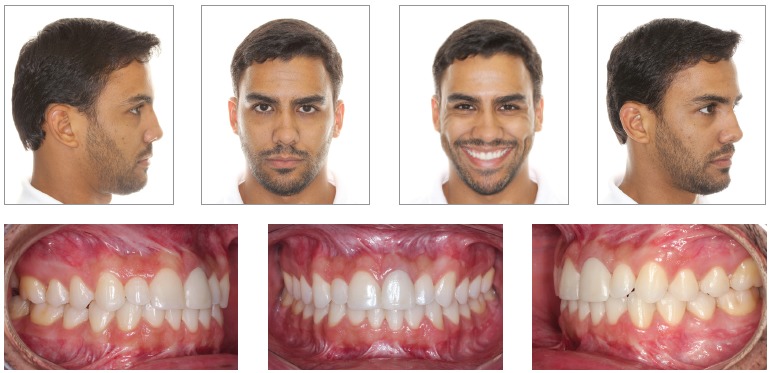
- Postoperative facial and intraoral photographs.

With segmental maxillary surgery, one can leave spaces between laterals and canines, while maintaining ideal occlusion, and subsequently enhance laterals with direct restorations or ceramic fragments (Figs 5, 6, 7). This will favor the predominance of maxillary central incisors, the smile arc and smile esthetics. The location of interdental osteotomy between canines and premolars, or between central incisors, might correct the transverse maxillomandibular relationship, but not the Bolton discrepancy.^10^

### Controlling incisor buccolingual angulation

Buccal protrusion of incisors is more common in patients with maxillary hypoplasia, and can be corrected by means of the following: premolar extraction or selective stripping and retraction; distal movement of posterior teeth; surgically assisted rapid maxillary expansion or surgical expansion through segmental maxillary osteotomy in three segments.

**Figure 8:**
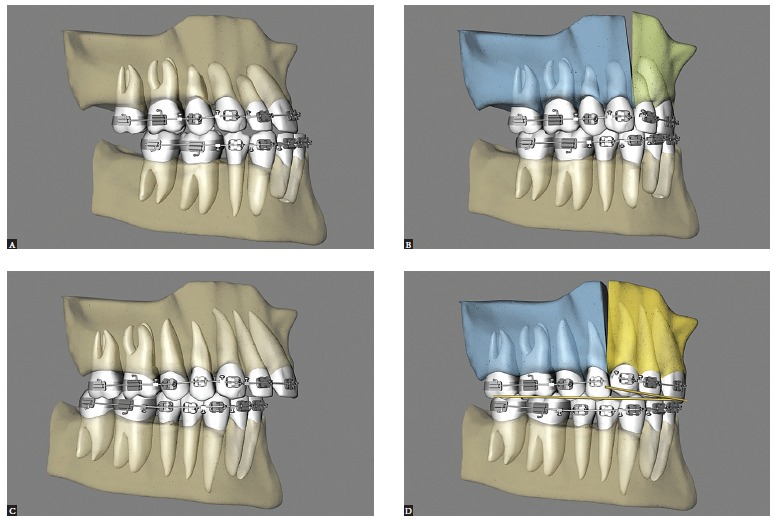
- Lateral view illustrative of clinical conditions in which incisor buccolingual angulation can be modified with the surgical technique. A) Preoperative condition with missing premolars, root resorption and incisors with increased buccolingual angulation. B) Three-segment maxillary surgery with correction of incisor buccolingual angulation. C) Preoperative condition showing increased incisor buccolingual angulation. D) Three-segment maxillary surgery with correction of incisor buccolingual angulation, but inadequate canine occlusion.

Segmental surgery between laterals and canines provides the best control in uprighting the segments, as compared with segmentation between canines and premolars. Canines are transitional teeth from the anterior and posterior segments, and therefore have torques that differ from those of the incisors. So one can, for example, upright the incisors without being affected by the canines. If this were performed with the technique between canine and premolar, canines would lose their ideal occlusion due to contact with the mesial surface of premolars, or infraocclusion^10^position (Fig 8). 

###  An easier technique

This technique is more easily performed than osteotomy between canines and premolars, since the location is more anterior and the bone is less thick in the region.^9^

## ADVANTAGES OF SEGMENTAL MAXILLARY OSTEOTOMY

When maxillary surgery is performed in multiple segments, it includes, in addition to the sagittal and vertical planes, the transverse plane as well. The tridimensional control afforded by these segments ensure better esthetic and functional results.^6^ The advantages of this technique are described in Table 2.

**Table 2:** - Summary of advantages and disadvantages of segmental maxillary osteotomy.

**Advantages**	**Disadvantages**
1- Single-stage surgery	When there are already 2 occlusion planes between canines and premolars it is not possible to segment between the lateral and canine
2- Intraarch asymmetry correction	
3- Controlling the Curve of Spee	When there is maxillary anteroposterior skeletal excess and premolar extractions are planned it is not possible to segment between lateral and canine
4- Controlling the Curve of Wilson	

### A single surgical stage

Segmental maxillary surgery involving three segments enables correction of the vertical, sagittal and transverse planes at the same surgical time;^6^ whereas surgically assisted maxillary expansion is a technique that corrects the transverse relationship only. The diastema between central incisors and the expansion screw will be present for six months prior to the installation of fixed orthodontic appliances. This will entail a longer orthodontic treatment and longer surgical time.^10^

### Correction of intra-arch asymmetry

**Figure 9:**
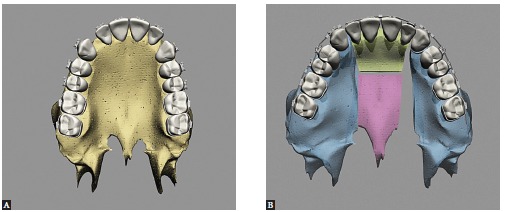
- Occlusal view illustrative of three-segment maxillary osteotomy: A) Preoperative condition, showing maxillary asymmetry. B) Postoperative condition with asymmetrical space closure between canines and laterals, and further expansion of the left hand side for asymmetry correction.

The osteotomized segments can be manipulated independently, thereby allowing tridimensional corrections to be implemented. This does not occur in surgeries involving a single segment. Intra-arch asymmetries can be corrected by asymmetric manipulation of segments, such as closing or creating spaces (Fig 9).

### Controlling the Curve of Spee 

An accentuated curve of Spee of the maxilla is more common in patients with a high occlusal plane and anterior open bite. This condition hinders the development of a good dental intercuspation. Careful evaluation of this curve is important given that if correction is performed with orthodontic mechanics alone, it may not be stable.^10^

**Figure 10:**
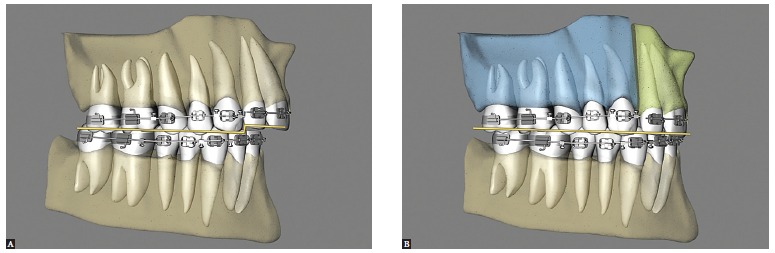
- Lateral view illustrative of three-segment maxillary surgery, with leveling of the Curve of Spee. A) Preoperative condition. B) Postoperative condition.

**Figure 11:**
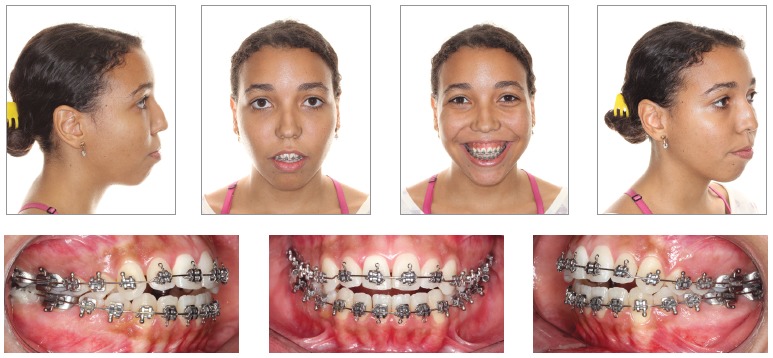
- Preoperative facial and intraoral photographs.

**Figure 12:**
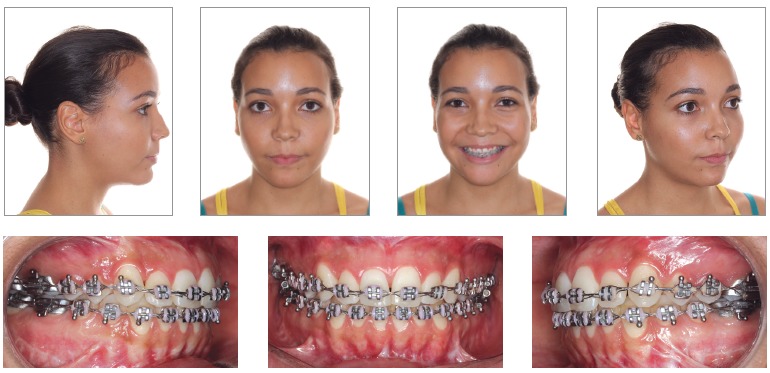
- Postoperative facial and intraoral photographs.

The anterior and posterior segments of the maxilla can be leveled individually with orthodontic mechanics by establishing different levels for anterior and posterior teeth. Leveling will then be performed during maxillary surgery in three segments, which will enable the correction of the accentuated curve of Spee and a better occlusion (Figs 10,11, 12).

### Controlling the curve of Wilson

**Figure 13:**
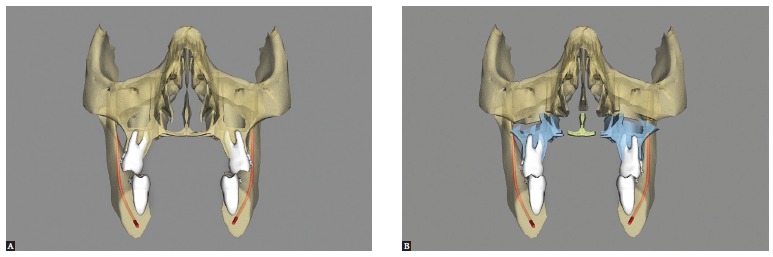
- Lateral view illustrative of three-segment maxillary surgery, with correction of the Curve of Spee: A) Preoperative condition. B) Postoperative condition.

If the occlusal surfaces of maxillary teeth are inclined labially, it may become difficult to achieve an appropriate occlusal relationship. In the presence of a transverse maxillary deficiency, an accentuated curve of Wilson and posterior crossbite, an orthodontic or orthopedic correction, or even an approach with surgically assisted maxillary expansion, will be inappropriate, since this curve will be rendered even more accentuated with these mechanisms. In these cases, surgical expansion by means of segmental maxillary osteotomy may be indicated to decrease the curve of Wilson and improve the final occlusion^10^ (Fig 13).

## DISADVANTAGES OF SEGMENTAL MAXILLARY OSTEOTOMY BETWEEN LATERALS AND CANINES

Segmental maxillary surgery between laterals and canines has some disadvantages when compared with surgery between canines and premolars in the cases presented in Table 2. 

### Presence of two occlusion planes in the maxilla between canines and premolars

**Figure 14:**
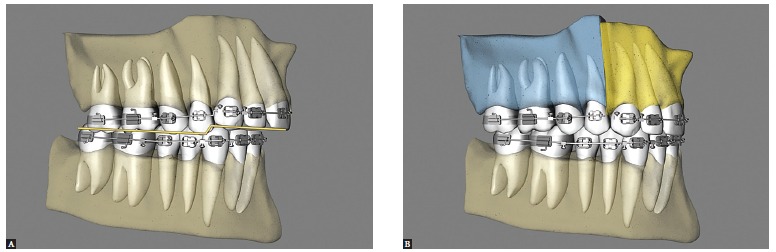
- Lateral view illustrative of three-segment maxillary surgery, with correction of the Curve of Spee, enlarged in the maxilla. A) Preoperative condition. B) Postoperative condition.

The first disadvantage is when two occlusion planes are already present in the maxilla, and their transition is between canines and premolars. Thus, one of the goals of preoperative orthodontic treatment would be leveling the maxilla in three segments: one anterior, from canine to canine, and two posterior, from premolars to second molars. The leveling of these curves would be carried out surgically^9^ (Fig 14). 

### Anteroposterior skeletal excess of the maxilla

**Figure 15:**
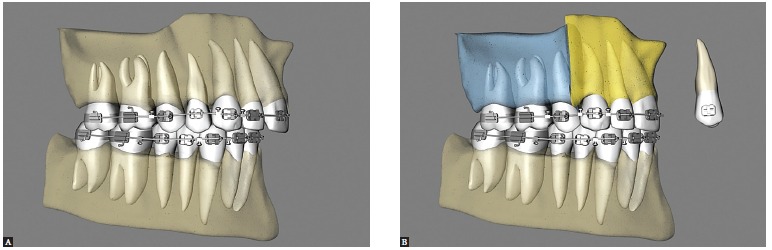
- Lateral view illustrative of three-segment maxillary surgery, with correction of maxillary skeletal protrusion. A) Preoperative condition. B) Postoperative condition with premolar extraction during the same surgery, and subsequent posterior premaxillary displacement to correct anteroposterior maxillary excess.

The second downside is when there is anteroposterior skeletal excess of the maxilla. One can plan bilateral premolar extractions by segmenting the maxilla in this region, and then move the canine-to-canine block posteriorly, thus achieving a better, more esthetic and functional outcome^9^ (Fig 15). 

## SEGMENTAL MAXILLARY OSTEOTOMY SEQUENCE

A mucoperiosteal maxillary buccal incision is performed, with the purpose of exposing the maxilla, above the attached gingiva and the tooth apices, extending from the mesial of first molars from one side to the contralateral side. Mucoperiosteal detachment is performed exposing the bone in the anterior maxillary region, with tunneling in the lateral region of the maxilla, thereby preventing laceration of the maxillary buccal pedicle and exposure of the buccal fat pad. A delicate detachment is necessary in the interdental region, between the roots of the lateral incisor and the canine, on each side of the nasal mucosa floor and medial wall of the nasal cavity and nasal septum perichondrium.

**Figure 16:**
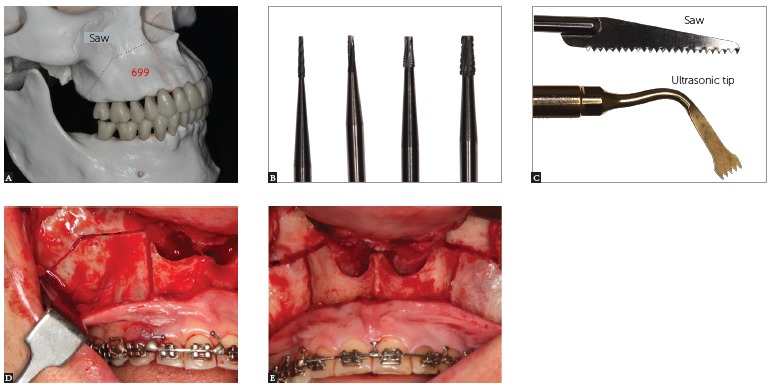
- A) Lateral view illustrative of the maxilla, showing the design of Le Fort I osteotomy and interdental segmentation between lateral and canine; B) Fissure burs #699, #701, #702, and #703, with different thicknesses. C) Saw blade and ultrasonic tip. D) Lateral view of the maxilla, showing the design of Le Fort I osteotomy and interdental segmentation between lateral and canine. D) Front view of the maxilla, showing the design of Le Fort I osteotomy and interdental segmentation between lateral and canine.

A tool should be used to protect the nasal mucosa. Le Fort I osteotomy is carried out using a 701 fissure bur and reciprocating saw (Fig 16).

Interdental osteotomy of the maxillary cortex is performed with the aid of a 699 fissure bur (Fig 16B and Table 3) between the roots of lateral incisors and canines. 

**Table 3:** - Summary of features conducive to a successful segmental maxillary osteotomy.

**Successful segmental osteotomy technique**
1- Adequate space between the roots of laterals and canines (3 mm).
3- Burs #699 and ultrasonic tips.
2- Blood supply maintenance.
4- Carefully performed, atraumatic surgery.
5- Spatula osteotomes.

**Figure 17:**
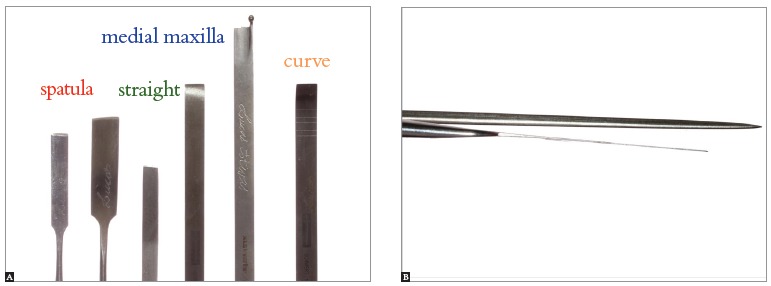
- A) Osteotomes used for segmental maxillary osteotomy. B) Differences in the thickness of spatula and straight osteotomes.

Use a spatula osteotome in the interdental osteotomies (with digital support in the palatal mucosa, detecting the presence of the instrument, thus avoiding damage to soft tissue); and the septum and curve, respectively, in the regions of the septum and pterygoid process of the maxilla (Fig 17).

Lowering of the maxilla is performed along with mobilization with a Rowe forceps, Seldin elevator, or Tessier lever.

If necessary, a septoplasty, turbinoplasty and suturing of the nasal mucosa can be performed at this time.

**Figure 18:**
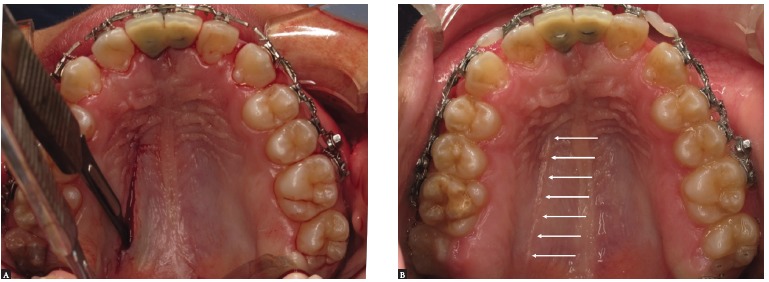
- A) Paramedian incision at 45^o^ of the palatal mucosa with slide #15. B) Healing of the palatal mucosa.

Palatal osteotomy is then performed using ultrasonic tips (Fig 16C) in the shape of an H (Table 3). The paramedian incision in the palatal mucosa can be performed between the raphe and the palatal artery, extending from the region of the first molar to the ipsilateral canine. It is important to position a scalpel blade #15 at a 45° angle. This is to ensure improved healing through broader connective tissue contact. This incision allows a transverse maxillary expansion greater than 10 mm while preventing a complication in the communication between the maxillary sinus and the oral cavity. Through this incision, the mucoperiostel detachment of the palate is performed, leaving the mucosa of the alveolar process attached^22^ (Fig 18).

**Figure 19:**
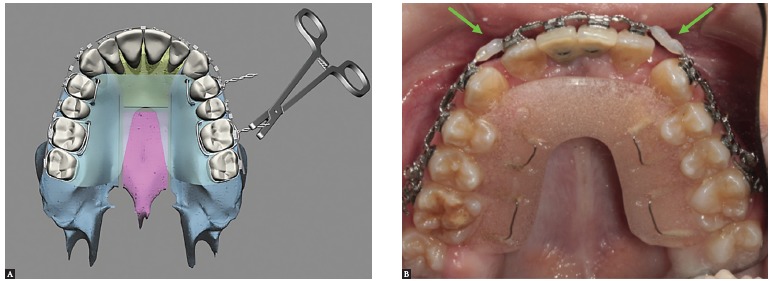
- A) Palatal guide being placed in the maxilla to impart stability to the segments. B) The palatal guide is kept in place for 60 days after surgery. Once occlusal stability is achieved and no premature dental contacts are present, the orthodontic archwires are joined with light-curing resin.

At this time, the three segments are mobilized, the palatal guide is inserted and the intermaxillary splint is present in the final occlusion (Fig 19).

**Figure 20:**
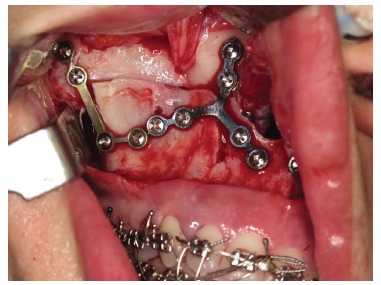
- Rigid internal fixation of the maxilla by means of plates ("T" and "L"), and 2.0 mm system screws.

Finally, internal rigid fixation is performed with the use of miniplates and system 2.0 mm titanium screws (Fig 20). This fixation follows the vertical planning of the maxilla obtained during surgery through external reference with a Kirschner wire. It is important, therefore, that the maxillary bone be free from bone interference and remain passive in its final position as planned.

**Figure 21:**
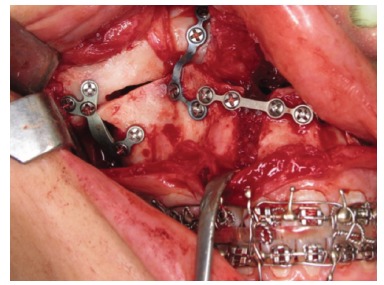
- Particulate bone grafting in the interdental osteotomy gaps.

After this fixation, autogenous bone grafts are used to improve skeletal stability, maintain the desired inclination of maxillary incisors, and provide primary bone healing in the regions of interdental gaps and maxillary step^23^^,^ 24 ^,^ 25 ​​(Fig 21).

The intermaxillary splint is then removed and, in centric relation, the relationship between the mandible and maxilla is examined to ensure the correct position of the latter.

Plication of the alar base is then performed, and the wounds sutured.

## STABILITY

Marchetti et al^26^ compared the stability of surgically assisted palatal expansion and segmental maxillary osteotomy two years postoperatively. Their results showed that segmental osteotomy for maxillary expansion yielded greater stability.

Krestscmer et al^27^ conducted a comparative study on the stability of Le Fort I osteotomy in one segment and three segments. The authors concluded that there was no statistical difference in bone relapse in multiplanar movements in these techniques. They reported that the decision to segment the maxilla must be made in accordance with the occlusal benefits obtained, and that the individual indications of each patient should therefore be taken into account.

Arpornmaeklon et al^12^ retrospectively analyzed the stability of maxillary advancement comparing a group subjected to Le Fort I osteotomy without maxillary segmentation (11 patients) with a group who underwent Le Fort I osteotomy with maxillary segmentation (15 patients). The analysis was performed with cephalometric radiographs obtained before surgery (T_1_), immediately after surgery (T_2_), and at least one year after surgery (T_3_). Results showed that the cases without segmentation experienced a higher relapse in both vertical and horizontal directions than cases with maxillary segmentation.

## COMPLICATIONS

The literature reports that the most frequent complications of segmental maxillary osteotomy are: necrosis of the repositioned maxillary segment, broadening of the alar base, nose tip rotation, and tooth devitalization, particularly canines.^8^ It further stresses the influence of the surgical technique of choice on the results.^28^ Other complications to consider are differences in the dentoalveolar region between anterior and posterior segments, bone loss and gingival margin degeneration.^29^

Sher^30^ sent out 135 questionnaires to oral and maxillofacial surgeons in the United States and Canada. The total number of segmented osteotomies was 6,195 of which 1,133 had been performed in the anterior maxilla. Complication rate was 0.32%, and the highest prevalence of complications were tooth mobility, injury and loss of teeth. The researcher suggested that to avoid complications, it is necessary to encourage the use of orthodontic mechanics at the expense of segmentations; avoid interdental osteotomies, if the space between roots is insufficient; and use osteotomes instead of saws. He concluded that factors such as surgeon experience, a shorter surgical time and proper postoperative follow-up can minimize complications (Table 3). 

Dorfman and Turvey^31^ documented changes in the level of the interdental bone crest after segmental osteotomies of the maxilla and mandible. The researchers inferred that a minimum space of 3 mm would be safe for performing interdental osteotomies between two adjacent teeth (Table 3). They also stated that the success of interdental osteotomies depends on maintaining an adequate blood supply to the osteotomized segments through planned incisions and minimal periosteal detachment in osteotomized segments (Table 3). 

Interdental osteotomies must be designed in conjunction with preoperative orthodontic treatment to ensure sufficient space to perform osteotomies.^32^ This is an important factor, since root divergence is critical to the success of segmental osteotomy.^22^ Performing interdental osteotomies in regions with restricted interradicular space is described as a risk factor for the development of marginal bone loss.^33^

## CONCLUDING REMARKS

Preoperative orthodontic goals can influence the achievement of suitable functional and esthetic results. Transverse maxillomandibular discrepancies of up to 4 mm, and those of dental volume or Bolton discrepancy, as well as changes in the buccolingual angulation and intra-arch asymmetry are occlusal problems that can be solved through orthodontic mechanics control. However, there are situations in which it is necessary to segment the maxilla, namely: transverse discrepancies greater than 4 mm, the presence of two occlusion planes and major root resorption.

Segmental maxillary osteotomy between laterals and canines is a versatile technique with several indications. Furthermore, it offers a host of advantages compared with single-piece osteotomy, or between canines and premolars. 

It is important to learn about its indications, limitations and surgical technique with proper manipulation of the gingiva and bone, thus avoiding transoperative and postoperative complications.

As shown above, the literature substantiates the stability and complications of segmental maxillary osteotomy, but few studies have reported these features of the technique when it is employed between laterals and canines. Further studies are warranted to throw more light on this technique by addressing stability, complications, surgical and orthodontic treatment time, the quality of functional and esthetic results, and regional epidemiological data.
